# Functional characterization of *Caenorhabditis elegans cbs-2* gene during meiosis

**DOI:** 10.1038/s41598-020-78006-w

**Published:** 2020-12-01

**Authors:** Pamela Santonicola, Marcello Germoglio, Domenico Scotto d’Abbusco, Adele Adamo

**Affiliations:** Institute of Biosciences and BioResources, National Research Council, Via Pietro Castellino 111, 80131 Naples, Italy

**Keywords:** Genetics, Genomic instability

## Abstract

Cystathionine β-synthase (CBS) is a eukaryotic enzyme that maintains the cellular homocysteine homeostasis and catalyzes the conversion of homocysteine to L-cystathionine and Hydrogen sulfide, via the trans-sulfuration pathway. In *Caenorhabditis elegans,* two *cbs* genes are present: *cbs-1* functions similarly as to human CBS, and *cbs-2,* whose roles are instead unknown. In the present study we performed a phenotypic characterization of the *cbs-2* mutant. The null *cbs-2* mutant is viable, fertile and shows the wild-type complement of six bivalents in most oocyte nuclei, which is indicative of a correct formation of crossover recombination. In absence of synaptonemal complex formation (*syp-2* mutant), loss of *cbs-2* leads to chromosome fragmentation, suggesting that *cbs-2* is essential during inter-sister repair. Interestingly, although proficient in the activation of the DNA damage checkpoint after exposure to genotoxic stress, the *cbs-2* mutant is defective in DNA damage-induced apoptosis in meiotic germ cells. These results suggest possible functions for CBS-2 in meiosis, distinct from a role in the trans-sulfuration pathway. We propose that the *C. elegans* CBS-2 protein is required for both inter-sister repair and execution of DNA damage-induced apoptosis.

## Introduction

The CBS protein is a key enzyme of the trans-sulfuration pathway that catalyzes the formation of cystathione, producing hydrogen sulfide (H_2_S) and water from homocysteine (Hcy)^1^. In humans, the deregulation of CBS and the associated alterations in H_2_S levels and/or Hcy leads to a wide range of diseases involving the immune, cardiological and central nervous systems. H_2_S plays significant roles in mitochondria metabolism and bioenergetics, impacting on the electron transport and stimulating its activity^2^.

Mutations in CBS genes are implicated in colon and ovarian cancer^3–5^ and infertility^6^. In particular, CBS is overexpressed in ovarian cancer and contributes to advanced cancer progression and chemoresistance. In fact, downregulation of CBS leads to hypersensitivity to cisplatin, a DNA damage agent that induces inter-strand cross-links (ICLs)^7^.

In *C. elegans* there are two CBS orthologs, *cbs-1* and *cbs-2*. It has been demonstrated that *cbs-1,* unlike *cbs-2*, is implicated in hydrogen sulfide metabolism^8,9^. Up to date, *cbs-2* function is still unknown.

The *Caenorhabditis elegans* germline is a powerful system for studying meiosis and DNA repair because of the spatio-temporal organization of nuclei into all prophase I stages^10^.

During meiotic prophase I the physiological double-strand breaks (DSBs) are induced by the protein SPO-11. In *C. elegans*, as in all the other species, the number of DSBs largely exceeds the final number of Crossovers (COs) and only one DSB per homologue pair will result in a CO event^11,12^. The residual DSBs can be repaired by different pathways such as: inter-sister repair, synthesis–dependent strand annealing or through other alternative mechanisms^13^. The COs occur between homologous chromosomes, in order to generate genetic diversity and promote accurate chromosome segregation during meiosis. Homologous recombination (HR) represents the main repair mechanism of meiotic DSB repair in *C. elegans,* although it has been shown that under particular conditions, also non-homologous end joining (NHEJ) repair can occur at meiotic DSBs^14,15^. Recent findings have demonstrated that theta mediated end joining (TMEJ) pathway participate in DNA repair in *C. elegans* germ cells^16^ and that also single strand annealing (SSA) pathway plays a role in DSBs repair during meiosis^17^.

Following DSB formation, RecA-like protein RAD-51 loads on single-stranded DNA, flanking the DSB site and catalyzes the invasion of the double-stranded template DNA molecule. In wild-type *C. elegans*, RAD-51 foci arise during zygotene and early pachytene and decrease in number during middle-late pachytene stages^18^. Differences in frequency and/or distribution of RAD-51foci are indicative of DNA repair defects. The repair of DSBs by HR requires the cooperation of different factors, including those implicated in the formation of the Synaptonemal Complex (SC)^18,19^, as well as those implicated in the sister chromatid-mediated recombination, such as the ortholog of the mammalian BRCA1, BRC-1, and the ortholog of the mammalian FANCD2, FCD-2. In fact, mutations in these genes impair HR-mediated DSBs repair^20–22^.

By the end of pachytene, about half of the oocytes in *C. elegans* gonad undergo physiological cell death^23^. Additional exogenous or endogenous DNA damage trigger the DNA-damage checkpoint leading to increased germline apoptosis^20,21,24–26^.

The presence of DNA damage produces cell cycle arrest at G2 phase in the premeiotic proliferative region of gonad, inducing the activation of CEP-1 protein^27,28^. CEP-1 in turn induces EGL-1 expression, which disrupts the association between CED-4 (Apaf1) and CED-9 protein (Bcl2). This disruption induces the accumulation of CED-4 at the nuclear membrane^29,30^ and its re-localization to the nucleus periphery. The CED-4 accumulation activates the caspase CED-3^31–33^. Apoptosis culminates in the engulfment and degradation of the apoptotic cell^34^.

In this paper, we investigate the role of *cbs-2* gene in *C. elegans.* We first determined the phenotypic effects of *cbs-2* depletion, revealing *cbs-2* function in meiosis and in DNA damage apoptosis.

## Results

### *C. elegans cbs-2* mutant is hypersensitive to DNA damage

**Figure 1:**
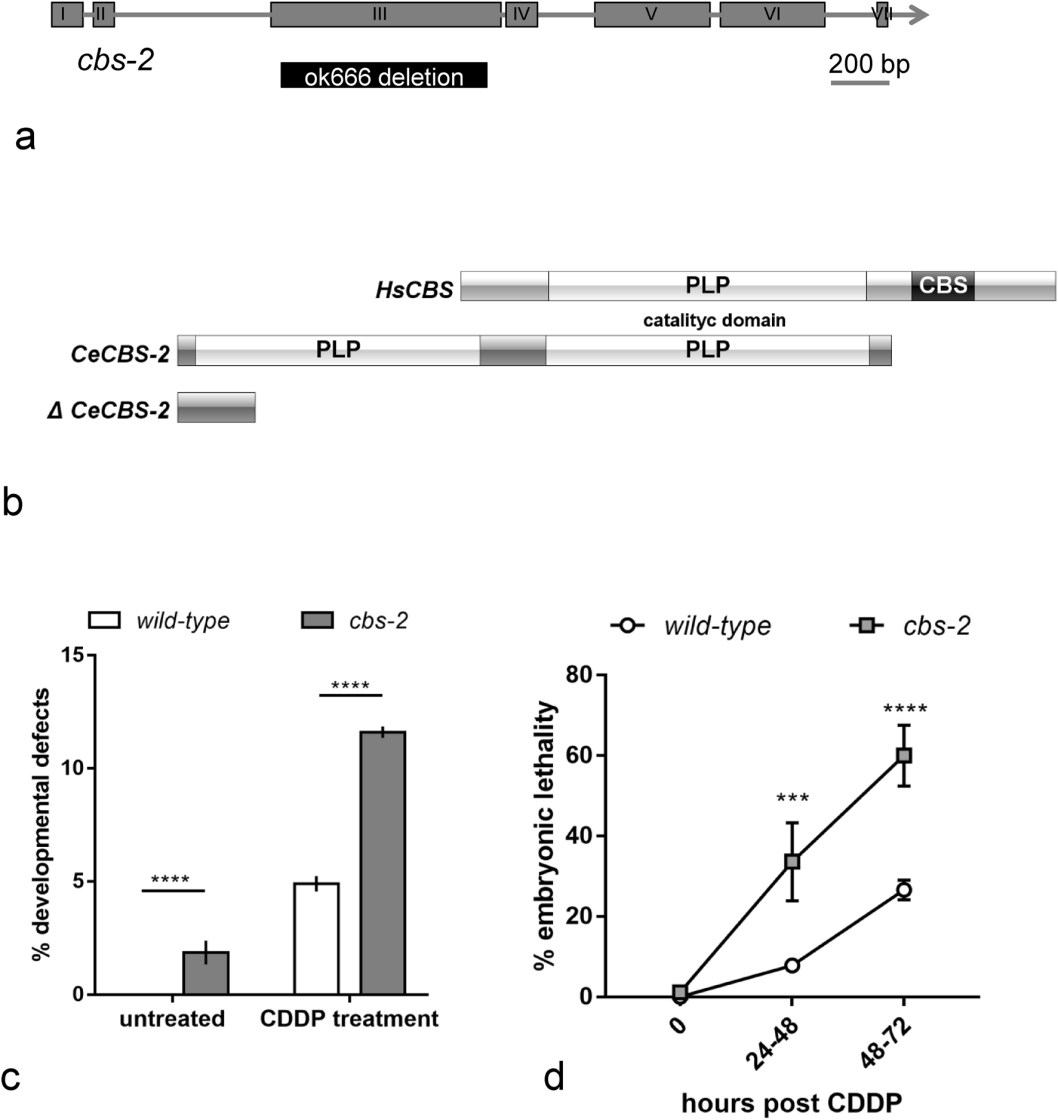
*cbs-2* mutant exhibited hypersensitivity to DNA damage. (**a**) Schematic representation of *cbs-2* locus. Boxes and straight lines represent the exons and introns, respectively. The *ok666* deletion (black box) depletes part of the third exon causing an early stop codon. (**b**) The primary structures of *Homo sapiens* CBS (HsCBS) (UniProt entry P35520) and *C. elegans* CBS-2 (CeCBS-2) (WormBase entry CE47819) are aligned by the PLP-domain. CeCBS-2 does not contain heme domain and the CBS cores. ΔCeCBS-2 contain only 73 N-terminal amino acids. (**c**) Developmental defects before and 24–48 h after 180 µM CDDP treatment. Error bars correspond to S.E.M. Experiments have been performed in triplicate. χ^2^ test of developmental defects *wild-type vs cbs-2:* untreated ****p < 0.0001, CDDP treatment ****p < 0.0001. (**d**) Embryonic lethality at different time points after treatment with 180 µM CDDP for *wild-type* and *cbs-2* mutant. Experiments have been performed in triplicate. Error bars correspond to S.E.M. χ^2^ test of dead embryos, *wild-*type *vs cbs-2*: 0 h, ***p = 0.0019; 24–48 h after CDDP treatment, ****p < 0.0001; 48–72 h after CDDP treatment, *p* < 0.0001.

The F54A3.4 (*cbs-2*) gene is the homolog of human CBS (UniProt entry P355320). The *cbs-*2 gene contains seven exons (Fig. 1a) and the predicted encoded protein exhibits 55% sequence identity with human CBS^9^. Its catalytic core contains the pyridoxal phosphate binding domain (PLP) followed by CBS core domain^35^. Protein domain prediction obtained by using InterPro^36^, revealed that CeCBS-2 contains two PLP domains and lacks the CBS core at C-terminal (Fig. 1b). Moreover, protein sequences alignment highlighted a greater homology between the C-terminal PLP domain of CeCBS-2 and HsCBS PLP domain (Fig. 1b and S1).

To determine whether *cbs-2* gene is transcribed, we analyzed, by quantitative RT-PCR, the absolute levels of *cbs-2* transcript and of *pmp-3,* used as “housekeeping gene”, in *wild-type* worms. The average of the CT (cycle threshold) obtained from three biological replicates were 27.40^+/−0.27^ and 23.96 ^+/−0.17^, respectively. Since CTs ≤ 29 were strong positive reactions and are indicative of abundant target nucleic acid in the sample, we moved onto analyzing the *cbs-2* mutant.

The *cbs-2(ok666)* allele presents a deletion of 692 bp in the third exon (Fig. 1a). The putative amino acid sequence of ΔCBS-2 obtained by ExPASy translate tool shows the presence of an early stop codon (Fig. 1b and S2).

Viability assessment of *cbs-2* mutants revealed increased embryonic lethality as well as occasional postembryonic developmental defects in a frequency that was statistically different from wild-type, and that might be a consequence of defects in the repair of DNA damage (Table 1, Fig. 1c and Table S1).

**Table 1:** Screening of *wild-type* and *cbs-2* mutant.

	*wild-type*	*cbs-2*
Parental	15	20
Laid eggs	4006	3602
Dead embryos	20	42
Hatched eggs	3986	3560
Males	2	5
Average brood size	267	180
Dev. def	1	65
Dead embryos (%)	0.5	1.17
Males (%)	0.05	0.14
Dev. Def. (%)	0.025	1.83

χ^2^ test dead embryos *wild-type vs*. *cbs-2* mutant: *p* < 0.0001. χ^2^ test developmental defects *wild-type vs.cbs-2* mutant: *p* < 0.0001.

We investigated whether CBS-2 could be involved in DNA damage repair by analyzing the sensitivity of *cbs-2* depleted worms to *cis*-platin (CDDP), a DNA damage inducing agent that causes ICLs.

Upon exposure to CDDP we observed increased embryonic lethality in *cbs-2* mutant compared to *wild-type*. After 24–48 h of CDDP treatment, the percentage of dead embryos in *cbs-2* mutant was five times higher than what we observed in *wild-type* (33.6% and 7.85% respectively), and 60% in *cbs-2* mutant and 26.6% in *wild-type* after 48–72 h of CDDP treatment (Fig. 1d).

Moreover, after 24–48 h of CDDP treatment, *cbs-2* mutant showed also a significant increase in the percentage of worms carrying developmental defects (11.6%) compared to *wild-type* (4.9%) (Fig. 1c, Table S1).

Taken together, these results support the hypothesis that the observed phenotypes in absence of *cbs-2* gene are due to defects in the DNA damage repair.

### *cbs-2* mutant is defective in inter-sister DNA repair during meiosis I

Taking advantage of the *C. elegans* germline, we investigated the proficiency of *cbs-2* mutants in repairing endogenous DNA damage, by analyzing key meiotic steps: repair of recombination intermediates and consequential cross-over formation. Chromosome pairing is coupled with the assembly of the SC and is followed by the formation of multiple physiological DSBs induced by the SPO-11 protein, which trigger meiotic recombination. RAD-51 recombinase binds single-stranded regions adjacent to resected DSBs forming distinct foci, which follow precise kinetics of loading onto and release from the chromatin during meiotic prophase I. Hence, analysis of RAD-51 foci formation and disappearance is widely used as a cytological marker for progression and resolution of recombination intermediates^18,20,21,37^. Mutants affecting homologous recombination repair of meiotic DSBs show altered levels and/or distribution of RAD-51 foci and irregular chromatin aggregates at diakinesis stage.

**Figure 2:**
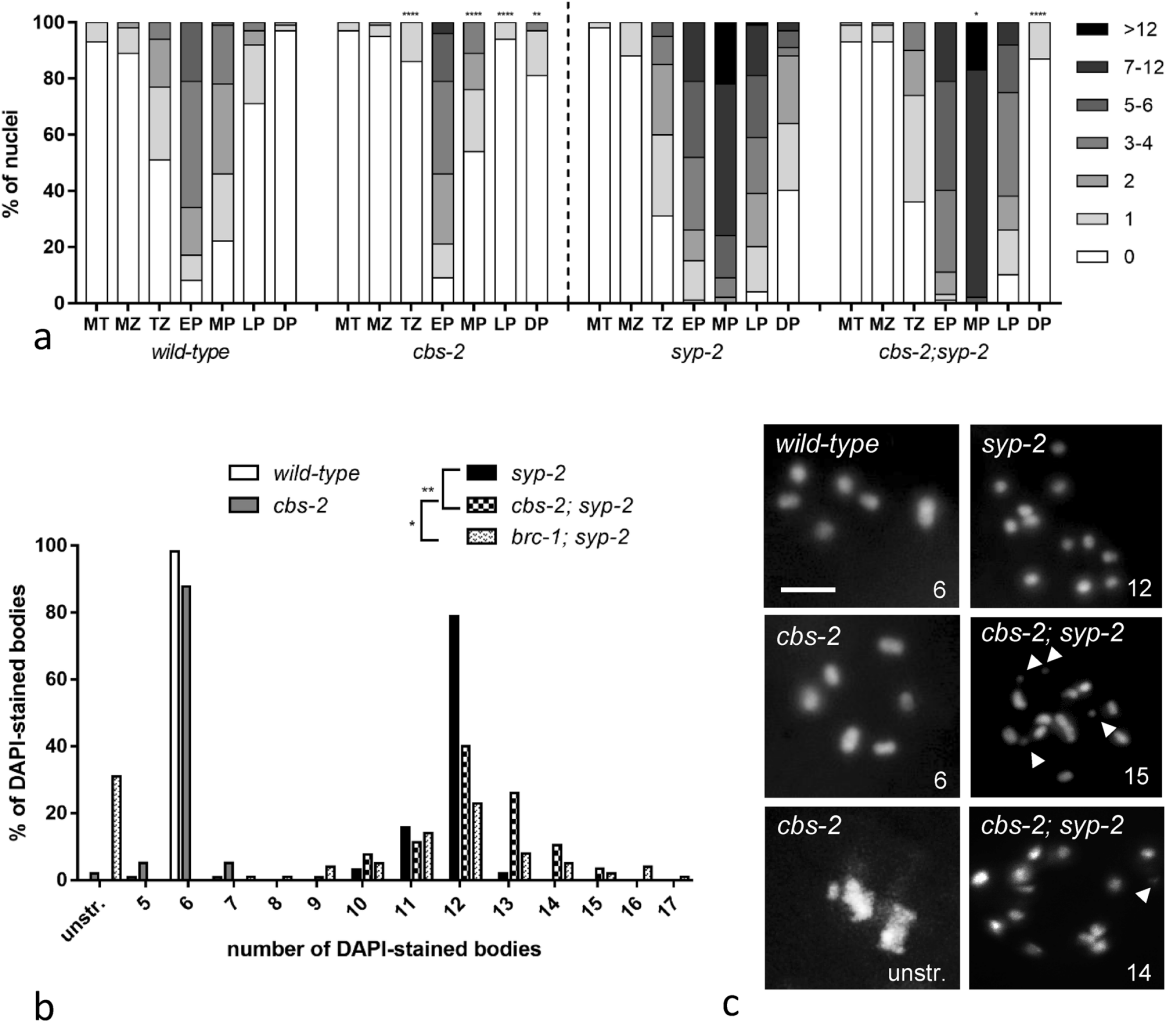
*cbs-2* mutant show defects in meiosis I. (**a**) Histograms represent quantification of RAD-51 foci in germline of the indicated genotypes. The y-axis represents the percentage of nuclei with the indicated number of foci. The x-axis represents the position (zone) along the germline. For each genotype an average of 100 nuclei from at least 4 different animals had been scored for mitotic tip, mitotic zone, transition zone, early, middle and late pachytene, while an average of 30 nuclei from at least 4 different animals had been scored for diplotene. Mann–Whitney *t-*test: *p = 0.05; **p = 0.002; ****p < 0.0001. Statistical analysis indicated in Table S2. (**b**) Quantification of DAPI-stained bodies in diakinesis oocytes of the indicated genotype (see colour legend on top of the graph). The y-axis reports the percentage of nuclei in each class and the x-axis indicates the number of DAPI-stained bodies. Number of observed diakinesis nuclei: *wild-type* = 114, *cbs-2* = 99, *syp-*2 = 95, cbs*-2;syp-2* = 115, brc-1;syp-2 = 102. Mann–Whitney *t*-test: *wild-type vs cbs-2 p* = 0.4724; *syp-2 vs cbs-2;syp-2* **p = 0.002; *cbs-2;syp-2 vs brc-1;syp-2* *p = 0.010. (**c**) Representative images of diakinesis nuclei of the indicated genotype stained with DAPI. The number of DAPI-stained bodies is indicated at the bottom right of each panel. Scale bar is 2 µm. Chromosome fragmentation is indicated by the white arrowheads.

The distribution of RAD-51 foci in *cbs-2* mutant appeared similar to *wild-type*, however the frequency of RAD-51 foci appeared significantly different compared to wild-type strain. Indeed, the number of RAD-51 foci in *cbs-2* significantly decreased in transition zone, middle and late pachytene whilst significantly increased in diplotene (Fig. 2a, Table S2).

At diakinesis stage, each condensed chromosome pair forms a compact body, which can be easily visualized by DNA staining (DAPI); diakinetic nuclei normally contain six DAPI-stained bodies. In *cbs-2* mutant most of the nuclei showed six DAPI-stained bodies corresponding to the expected six bivalents, although a low percentage of oocyte (2%) showed poorly condensed chromatin (Fig. 2b,c). HR in *C. elegans* leads to the formation of one inter-homolog CO event per each chromosome. The rest of the SPO-11 induced DSBs are repaired by non-CO mechanisms, using sister chromatid-mediated recombination, a process dependent on *brc-1,* ortholog of the mammalian BRCA-1 gene^20,38^.

The absence of the SC, such as in *syp-2* mutant, leads to a strong reduction of crossover recombination. Furthermore, in absence of SC, RAD-51 outbreaks disappear in late pachytene, impling that all DSBs are repaired, probably using the sister chromatid as a template^13,14,18^. In addition, in the *syp-2* mutant 12 achiasmatic chromosomes (univalent) are present in the diakinesis nuclei^39^, confirming that DSBs are repaired eventually. When inter-sister repair is inhibited, in absence of the SC, the meiotic DSB repair is impaired leading to both chromosomal fragmentation in diakinesis nuclei and persistence of RAD-51 foci at diplotene stage^13,15,40,41^. To determine whether *cbs-2* mutant is competent in repairing meiotic DSBs via inter-sister repair, we quantified DAPI bodies at diakinesis stage in *cbs-2; syp-2* double mutants as well as the number of RAD-51 foci, comparted to *syp-2* mutant.

As shown in Fig. 2b, in *cbs-2; syp-2* double mutants 40% of the diakinesis nuclei displayed ≥ 13 DAPI bodies (Fig. 2b,c), a phenotype reminiscent of that observed in the *brc-1; syp-2* double mutant (Fig. 2b,c).

This data suggests that CBS-2 may play a role during inter-sister repair.

Unexpectedly, RAD-51 staining was dramatically reduced in the diplotene stage in *cbs-2; syp-2* compared to *syp-2* mutant (Fig. 2a, Table S2). Never the less, diplotene RAD-51 reduction resulted to be similar to that observed in *brc-1; syp-2* double mutant diplotene^20,42^ (Figure S3).

### *cbs-2* promotes execution of DNA damage-induced apoptosis

**Figure 3:**
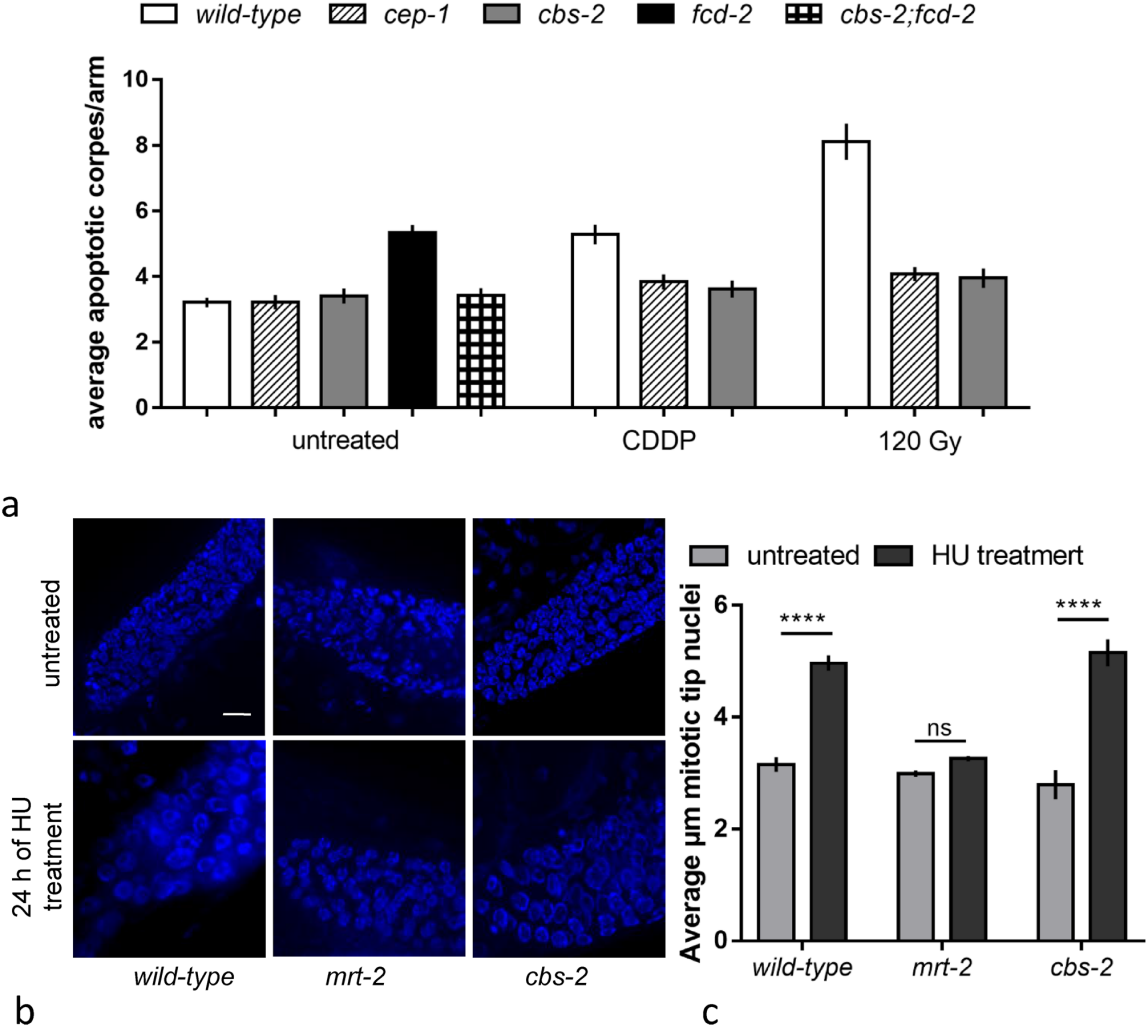
*cbs-2* mutant responds to genotoxic insults by activating DNA damage checkpoint but not DNA damage apoptosis. (**a**) Average apoptotic levels in the indicated genotypes. The y-axis shows the average number of SYTO-12-labeled nuclei per gonadal arm. Number of untreated gonads analysed: *wild-type* = 76; *cep-1* = 54; *cbs-2* = 89; *fcd-2* = 91; *cbs-2;fcd-2* = 50; number of analysed gonads after CDDP treatment: *wild-type* = 49; *cep-1* = 40; *cbs-2* = 111; number of analysed gonads after 120 Gy treatment: *wild-type* = 43; *cep-1* = 58; *cbs-2* = 67. Error bars correspond to S.E.M. Mann–Whitney *t*-test: *wild-type vs cbs-2 p* = 0.83, *fcd-2 vs cbs-2;fcd-2* ****p < 0.0001, *wild-type vs cbs-2* after CDDP treatment ****p < 0.0001, *wild-type vs cbs-2* after 120 Gy ****p < 0.0001. (**b**) Representative images of DAPI-stained nuclei in the mitotic tip of the germline before and after 24 h of HU treatment of the indicated genotypes. Scale bar 10 µm. (**c**) Average diameter of mitotic nuclei before and after 24 h of HU treatment. Error bars correspond to S.E.M. A number of 100 nuclei were scored for each column. One-way ANOVA Kruskal Wallis: *wt vs wt* HU treatment, ****p < 0.0001*; mrt-2 vs mrt-2* HU treatment; ns *p* = 0.07; *cbs-2 vs cbs-2* HU treatment, ****p < 0.0001; *wild-type HU treatment vs cbs-2* HU treatment, *p* = 0.277.

Under standard conditions of growth, about half of the oocytes in the *C. elegans* germline undergo physiological cell death by the end of pachytene^23^. The physiological germline apoptosis can be scored in vivo using SYTO-12 and an average of about 3 apoptotic nuclei in *wild-type* are observed^43^. In addition, accumulation of DNA damage can occur in the germline, either upon failure in faithfully executing meiotic recombination or in response to genotoxic stress, which activates the DNA damage checkpoint, resulting in cell cycle arrest at the G2 phase in order to allow DNA repair. When DNA damage levels are excessive, cells might undergo apoptosis and an increase in the SYTO-12-stained nuclei can be observed. In fact, mutants with defective DNA repair in *C. elegans*, as *brc-1* and *fcd-2* mutants, trigger the DNA-damage checkpoint leading to an increase in the apoptotic levels in the germline^20,21,24,25,44^. We investigated the apoptotic levels in the hermaphrodite germline of *cbs-2* mutant and unexpectedly, even if *cbs-2* mutant is defective in inter-sister repair, the apoptotic levels were not significantly different from *wild-type* animals (Fig. 3a). We therefore tested whether exogenous DNA damage, induced by CDDP treatment, was able to enhance apoptosis in *cbs-2* mutant. Although the apoptotic levels were significantly increased in *wild-type* after 48 h of CDDP treatment, no enhancement was observed in treated *cbs-2* mutant (Fig. 3a). We therefore confirmed our data inducing DSBs with another source of exogenous damage, γ-rays (irradiating worms at 120 Gy), and also in this case we did not observed any increase in the apoptosis levels (Fig. 3a).

To further investigate *cbs-2* role in DNA damage apoptosis, we tested whether endogenous DNA damage was proficient in inducing apoptotic cell death. We assayed the apoptotic levels of *cbs-2* mutant in a *fcd-2* mutated background. *fcd-2* mutant is defective in DNA repair and shows higher levels of germline apoptosis due to the activation of the pachytene DNA damage checkpoint^21^. The depletion of *cbs-2* reduced the apoptotic level of *fcd-2* mutant to wild-type levels (Fig. 3a). Thus, our data support a role for CBS-2 in eliciting the DNA damage apoptosis after endogenous and exogenous DNA damage.

### Activation of the DNA damage checkpoint in the mitotic and meiotic region of the germline is intact in the *cbs-2* mutant

Since we demonstrated *cbs-*2 involvement in eliciting DNA damage apoptosis, we next tested whether the DNA damage checkpoint is correctly activated in *cbs-*2 mutant. The nuclei residing in the tip of the *C. elegans* germline are engaged in mitotic proliferation and respond to DNA damage triggering a temporary block of cell cycle progression to allow DNA repair. As a consequence of cell cycle arrest, these nuclei appear enlarged. To establish whether *cbs-2* mutant is able to activate cell cycle arrest, we measured the diameter of the nuclei in mitotic zone before and after hydroxyurea (HU) treatment, compared to *wild-type* as positive control and to *mrt-2* as negative control^45^. As shown in Fig. 3c, HU treatment induced similar increase in the average diameter of mitotic nuclei in *cbs-2* mutant and *wild-type* animals. Instead, in *mrt-2* mutant the average diameter of the mitotic nuclei remained constant before and after HU treatment, as expected upon defective arrest of cell cycle. These data suggest that *cbs-2* is not implicated in cell cycle arrest activation (Fig. 3b,c).

### *cbs-2* is not required for the expression and localization of the proapoptotic factors CEP-1 and EGL-1 after DNA damage

**Figure 4:**
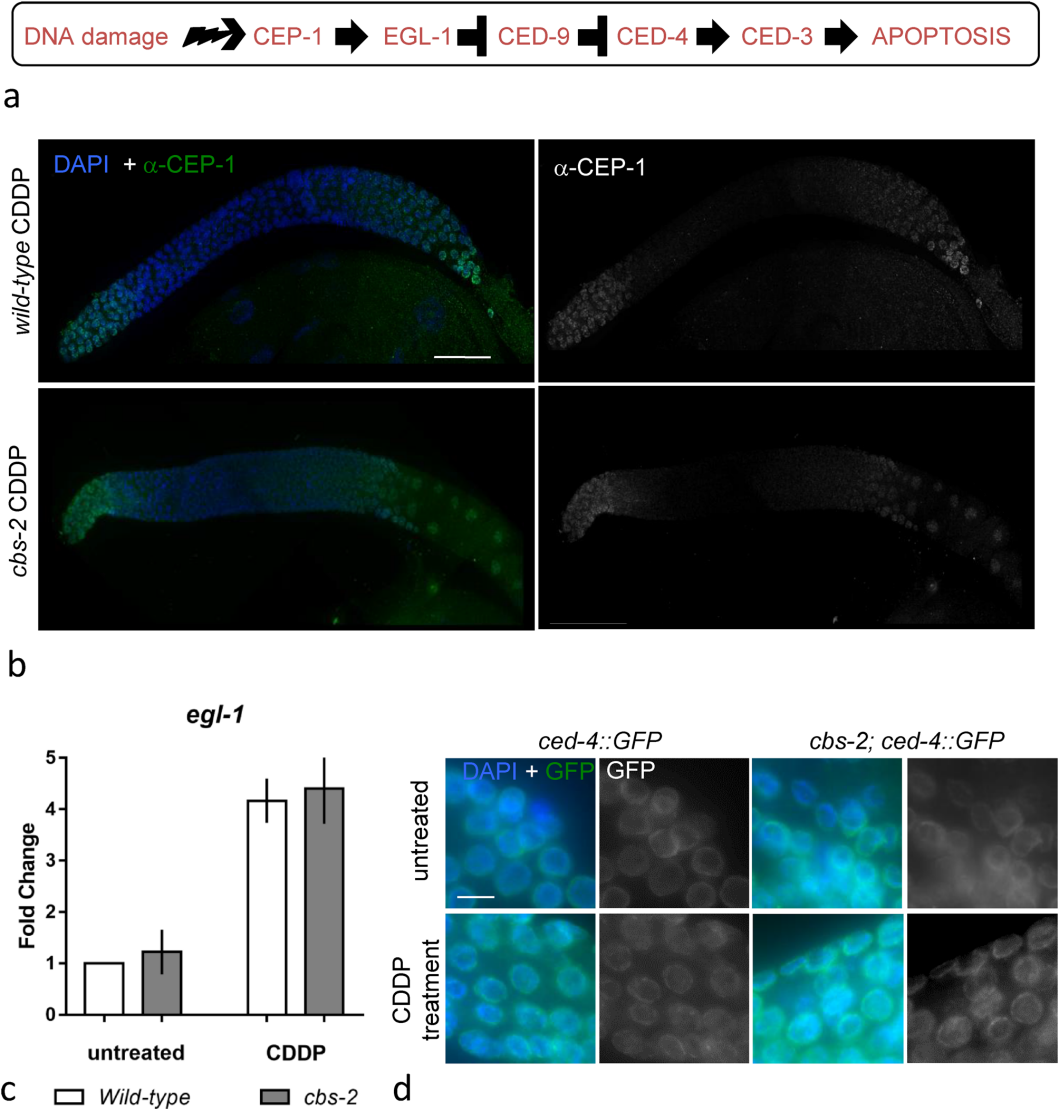
Loading of proapoptotic machinery is unperturbed in *cbs-2* mutant upon DNA damage. (**a**) Schematic representation of the DNA damage response in the *C. elegans* germline. (**b**) Immunolocalization of the CEP-1 protein in the germline of *wild-type* and *cbs-2* mutant 24 h after CDDP treatment. Left panels DAPI staining in blu (DAPI) + antibody against CEP-1 (αCEP-1) in green. Right panels staining with antibody against CEP-1 (αCEP-1) in white. Scale bar 20 µm. (**c**) Relative level of *egl-1* transcript by quantitative RT-PCR in *wild-type* and *cbs-2* mutant before and after CDDP treatment. *y*-axis indicates fold change. Error bars correspond to S.E.M. Experiments have been performed in triplicate. No significant difference in fold change was observed between *wild-type* and *cbs-2* mutant. (**d**) Representative images of late pachytene nuclei of the CED-4::GFP in *wild-type* and *cbs-2* mutant, under physiological conditions and after 24 h CDDP treatment. First and third panels DAPI stained nuclei in blu (DAPI) + CED-4::GFP (GFP) in green. Second and last panels CED-4::GFP (GFP) in white. Scale bar 5 µm.

We tested whether *cbs-2* mutated worms were competent in the expression and localization of the proapoptotic factors CEP-1 and EGL-1 after treatment with DNA damage agent (Fig. 4a).

*cbs-2* mutant showed wild-type CEP-1 loading in the mitotic and in the late pachytene nuclei 24 h after CDDP treatment (Fig. 4b). To assess the *egl-1* gene expression we analyzed, by quantitative RT-PCR, the relative *egl-1* transcript levels before and after DNA damage in *cbs-2* mutant and in *wild-type*. CDDP treatment induced similar increase in the expression levels of *egl-1* transcript. After CDDP treatment in both *wild-type* and *cbs-2* mutant, *egl-1* transcript levels increased four times (Fig. 4c). Thus, in absence of *cbs-2* gene, CEP-1 is competent to induce *egl-1* expression after DNA damage.

We can conclude that *cbs-2* is not involved in triggering the DNA damage checkpoint in meiotic and mitotic region of the gonad but it may have a role in the execution of the DNA damage apoptosis.

### CBS-2 is not required for proper localization of the apoptotic effector CED-4 after DNA damage

Since we demonstrated that the DNA damage checkpoint was correctly activated in *cbs-2* mutant after CDDP treatment, we then wondered whether the failure in DNA damage apoptosis execution might depend on altered localization of the apoptotic factors acting downstream EGL-1. We then assessed CED-4 localization, before and after DNA damage, using a transgenic strain expressing CED-4::GFP under the *ced-4* promoter^29^. As shown in Fig. 4d, GFP expression was detected in *cbs-2* mutant and wild-type untreated worms, moreover CDDP treatment induced an increase in GFP signal in the mitotic region and late pachytene regions in both strains (Fig. 4d), suggesting that the apoptotic response defects observed in *cbs-2* depleted worms is not due to the downregulation of CED-4.

## Discussion

Human CBS is known to catalyze the production of hydrogen sulfide and cystathione from Homocysteine. In *C. elegans* two *cbs* genes are present, *cbs-1* and *cbs-2.* Previous studies demonstrated that, unlike *cbs-1*, *cbs-2* is not implicated in hydrogen sulfide metabolism^9^. Using a protein domain prediction software we have shown that CeCBS-2 PLP domain have a high degree of conservation with HsCBS PLP domain and further, we found that *cbs-2* is regularly transcribed under physiological conditions of growth in wild type animals.

In this paper we determine the phenotypes caused by the absence of *cbs-2* gene in *C. elegans.*

The hypersensitivity to DNA damage, the increase in embryonic lethality and developmental defects, as well as the occasional presence of condensed chromatin in oocyte nuclei of *cbs-2* mutated worms (Figs. 1c,d, 2b,c) suggest an inefficient repair of recombination intermediates in meiosis. We demonstrate for the first time that *cbs-2* is implicated in the repair of meiotic DSBs using sister chromatid as template. This is based on the observation that *cbs-2* mutant in a *syp-2* mutated background showed 40% of diakinetic nuclei with a number of DAPI stained body higher than 13, instead of 12 achiasmatic chromosomes (univalents) (Fig. 2b,c). Furthermore, we have not observed RAD-51 foci persistence at diplotene stage in *cbs-2;syp-2* typical of the defective meiotic DSB repair (Fig. 2a, Table S2). This phenotype is similar to that observed for *brc-1* mutant (Fig. S3). It had been demonstrated that BRC-1 is essential for both inter-sister repair and recruitment/stabilization of the RAD-51 factor to DNA damage in absence of SC^20,42^. We speculate that CBS-2, as BRC-1, may play a role in recruitment and/or stabilization of RAD-51 recombinase to DNA damage during meiosis I.

Our findings are in line with a previous observation regarding the specific germline expression of *cbs-2*^46^. These results led us to speculate that in *C. elegans* the two CBS proteins, CBS-1 and CBS-2, act in different tissues. In fact, previous findings showed that CBS enzymatic function seems to be unaltered in *cbs-2* mutant^9^ suggesting a main role of CBS-1 in CBS enzymatic activity in the whole organism and a different specific function of CBS-2 in the germline.

In *C. elegans*, mutants with genetic defects in DNA repair display activation of the DNA damage checkpoint, which ultimately results in an increase of the apoptotic level in the germline^20,21^.

The physiological apoptotic levels in the germline of *cbs-2* mutant are similar to *wild-type* strain. Our results show that CBS-2 is required for the execution of germ-line apoptosis in response to DNA damage originated by defective meiotic repair, as well as by DNA damage agents (CDDP and IR) (Fig. 3a).

We demonstrate that the DNA damage checkpoints activation function properly in *cbs-2* mutated worms, as in fact, the HU treatment produces cell cycle arrest in the premeiotic proliferative region of gonad (Fig. 3b,c). Germ cells in absence of *cbs-2* gene showed a normal localization of CEP-1 as well as a correct increased in the expression levels of *egl-1* transcript after treatment with DNA damage agents (Fig. 4b,c). Moreover, germ cells in *cbs-2* mutant showed increased CED-4 localization after DNA damage, similar to wild type (Fig. 4d).

These data suggest that the failure in the activation of DNA damage apoptosis in *cbs-2* mutant does not depend on defective apoptotic machinery.

Other meiotic genes, as *msh-4, msh-5 ,zhp-3* and *rad-51* (isoform A) whose actions are essential for DNA damage-induced apoptosis during *C. elegans* gametogenesis, appear to act downstream of the pro-apoptotic signal^37,43^. Further studies of these processes are needed to identify the exact step in which CBS-2 regulate the DNA damage apoptosis.

Studying the roles and the functions of genes involved in the regulation of the DNA damage apoptosis and in DNA repair is essential to highlight new processes that can be targeted by novel therapeutic approaches in the treatment of diseases caused by incorrect DNA repair and inappropriate apoptosis regulation.

## Experimental procedures

### Strains

Nematodes have been cultured at 20 °C on NGM plates with Escherichia coli OP50 as food source according to standard methods^47^.

The following *C. elegans* strains were provided by the Caenorhabditis Genetics Center: *wild-type* strain Bristol N2; RB839 *cbs-2(ok666)* II; AV276 *syp-2(ok307)V/nT1*[unc-?(n754)let-? qls50] (IV;V); DW102 *brc-1*(tm1145) III; NB105 *fcd-2(tm1298)* IV; CB5348 *mrt-2 (e2663)* III; VC172 *cep-1(gk138)* I; XR6 [*ced-4p::CED-4::GFP*].

Deletions in the following mutants were genotyped during genetic crosses using PCR primers flanking the deletions listed in Table S3.

### Bioinformatics

Alignment of amino acid sequences of *C. elegans* CBS-2 (WormBase entry CE47819) and human CBS (UniProt entry P35520) was performed using Cobalt Constraint-based Multiple Alignment online software with default parameters^48^. Predictions of protein domains both *C.elegans* CBS-2 (WormBase entry CE47819) and human CBS (UniProt entry P35520) were obtained using InterPro online software^36^. The putative amino acid sequence of *C. elegans* ΔCBS-2 *(ok666)* was obtained using ExPASy translate tool^49^.

### Screening of laying worms

Worms were isolated and cloned during the L4 larval stage on Petri plates and left at 20 °C to lay eggs for 3 days. Worms were transferred every 12 h onto fresh plates until the deposition of non-fertilized oocytes. Each plate was monitored every for 24/72 h to analyze the following parameters among the progeny: embryonic lethality, presence of males, aberrant phenotypes and larval arrests^21,37^. The percentage of embryonic lethality is calculated as the ratio of unviable eggs over laid eggs. The percentage of males, aberrant phenotypes and larval arrests is calculated as the ratio of males/aberrant/larval phenotype over the hatched eggs.

### DNA damage sensitivity

#### CDDP

Young adult worms were transferred onto fresh seeded plates containing 180 μM cis-diamminedichloroplatinum(II) (CDDP, Sigma). Worms were picked after 24 h for cytological procedures or transferred onto fresh plates every 12 h to score embryonic lethality (unhatched eggs) and organisms with evident postembryonic developmental defects under a dissecting microscope.

#### HU

Young adult worms were transferred onto freshly seeded plates containing 40 mM Hydroxyurea (HU, Sigma) and picked after 24 h for cytological procedures.

#### IR

Young adult worms were transferred on freshly seeded plates and exposed to ionizing irradiation from a _137_Cs source for a time corresponding to 120 Gy. Animals were scored 24 h after treatment.

### Real time-PCR

RNA was purified from mixed population of animals either untreated or 48 h post 180 μM cisplatin, using the QIAGEN (Valencia, CA) mini-kit according to the manufacturer’s instructions. Residual DNA present in the RNA preparations was removed by using the DNA-free DNA Removal kit (Ambion). RNA quality was checked by 2% agarose gel electrophoresis. cDNA was prepared from 2 µg of total RNA by using a high-capacity cDNA Reverse Transcription kit (Applied Biosystem, Foster City, CA). For real-time quantitative PCR, 5 µl of the 1:10 dilution of cDNA was used in each reaction; the Power SYBR Green Master Mix (Applied Biosystems) was used^37^. RT-PCR results were recorded as relative gene expression changes after normalizing for *pmp-3* gene expression and computed using the comparative CT method (2–ΔΔCT) as previously described^50^. The 2-ΔΔCT value was > 1 for gene more highly expressed and < 1 for genes lowly expressed. RT-PCR data are the mean ± SD of three biological replicates. Primers used for gene expression analysis listed in Table S3.

### Immunostaining of meiotic nuclei

Gonads from 20 h post L4 worms were dissected in M9 solution (0.3% H2PO4, 0.6% Na2HPO4, 0.5% NaCl and 1 mM MgSO_4_). Slides were freeze-cracked in liquid nitrogen, then immersed at -20 °C in methanol, methanol/acetone (1:1) and acetone respectively for 5 min, followed by three washes in PBS for 5 min each time. Slides were blocked in 0.3% BSA in PBS for 30 min at 37 °C in a humid chamber. Primary antibodies were diluted in Ab buffer (1% BSA, 0.1% Tween-20, 0.05% sodium azide in 1X PBS). Slides were incubated for 90 min at room temperature followed by three washes in PBS for 5 min. Primary antibodies used in this study were: goat anti-CEP-1 (1:250 dilution)^25^, goat anti-GFP (1:150 dilution, Invitrogen); rabbit anti-RAD-51 (1:200 dilution)^18^. Appropriate secondary antibodies were conjugated with donkey anti-goat Alexa Fluor 488 or goat anti-rabbit Texas Red (1:400 dilution, Invitrogen). Slides were incubated for 60 min in the dark at room temperature, followed by three washes in PBS + 0.1% Tween-20 for 5 min each time. Slides were mounted with Prolong Gold Antifade reagent with DAPI (Life Technologies).

### Quantitative analysis of germline apoptosis

Adult nematodes were suspended in M9 solution and stained by incubation with 33 µM SYTO-12 (Molecular probes) for 1 h and 30 min at room temperature in the dark. The worms were then transferred to seeded plates to allow stained bacteria to be purged from the gut. After 30 min, the animals were mounted on 2% agarose pads in 2 mM levamisole^21^. The estimation of apoptotic levels for each genotype was calculated as the average number of apoptotic nuclei per gonadal arm^22^.

### Analysis of DAPI-stained germlines

Adult nematodes were suspended in M9 solution on glass slides, permeabilized and fixed with 10 µl of 100% Et-OH. Finally, slides were mounted in 10 µl of DAPI (2 ng/µl) diluted in M9. Numbers of scored nuclei are indicated in the legends of charts.

### Image collection and processing

Collection of images was performed using a Leica DM6 fluorescence microscope, Hamamatsu camera under the control of Leica LAS AF 6000 software. Images were processed using Leica LAS AF 6000 software and Image J/Photoshop programs. Quantitative analysis of RAD-51 foci and DAPI-stained bodies along the germline were performed on z series. Optical sections were collected at 0.18 µm and 0.50 µm increments respectively.

The quantitative analysis of RAD-51 foci was performed by dividing the germline into seven 36 × 36 μm zones (mitotic tip, mitotic zone, transition zone, early pachytene, middle pachytene, late pachytene diplotene stage), beginning from the distal tip^18^.

### Statistical tools

Statistical analyses for independent samples were computed through Mann–Whitney *t*-test. The level comparison of aberrant phenotypes and larval arrests of the different genotypes were computed through χ^2^ test. The statistical analyses of diameter of mitotic nuclei before and after treatment were computed through Mann One-way ANOVA Kruskal Wallis.

## Supplementary information

Supplementary Figures.
