# Supplementary material for: Functional characterization of Caenorhabditis elegans cbs-2 gene during meiosis
Source: Sci Rep. 2020 Dec 1;10:20913. doi: 10.1038/s41598-020-78006-w (PMC7708620; doi:10.1038/s41598-020-78006-w)
Supplement: Supplementary file 1 — Supplementary Figures. [file 41598_2020_78006_MOESM1_ESM.docx]

**Functional characterization of *Caenorhabditis elegans cbs-2* gene during meiosis**

# Pamela Santonicola^*^, Marcello Germoglio^*^, Domenico Scotto d’Abbusco^*^ and Adele Adamo^*^

CeCBS-2 1 MCLPTIKAFKTILDAGSHRATQLVPLKKLKAEFQLKSDIYVKLEYLNISGSLEDRAAHRAFEMSNGIQRGDEVYVTAGGS 80

HsCBS --------------------------------------------------------------------------------

CeCBS-2 81 SAVSYATVAAVKGVRLTAHAPPGAFDQVRTILSTLGTKIVELPVSNFREARLQADLLAKRRGVYNLDGICEKAAFLANLE 160

HsCBS --------------------------------------------------------------------------------

CeCBS-2 161 GTAPEIEKAGKTSAVVVPLDAGGASAAAGIAAYFLKDQKTTVVGVRSSASASASAPAPETEVLLQKYKVEIRDAPESYTF 240

HsCBS --------------------------------------------------------------------------------

CeCBS-2 241 TRHLIETEGIMAGPSSGAAVLEAIKLAKDLPAGSVVVVVLMDGIRDYLDADWMKVNGKKPI-KPREIFDPKVLDYDP--- 316

HsCBS 1 -----------------------------MPSETPQAEVGPTGCPHRSGPHSAKGSLEKGSPEDKEAKEPLWIRPDAPSR 51

CeCBS-2 317 -TKMVGEWKSSSKFR----PERPLVLDSVLDAIGKTPLVKLQHVPKAHGVRCNVYVKCEFLNAGGSTKDRIAKKMVEIAE 391

HsCBS 52 CTWQLGRPASESPHHHTAPAKSPKILPDILKKIGDTPMVRINKIGKKFGLKCELLAKCEFFNAGGSVKDRISLRMIEDAE 131

CeCBS-2 392 KTGKPGALTPGATTLIEPTSGNTGIGLSLVAAVRGYKCLITMPEKMSKEKSTTLSVLGSTIVRTPNEAAFNSPSSHIGVA 471

HsCBS 132 ---RDGTLKPG-DTIIEPTSGNTGIGLALAAAVRGYRCIIVMPEKMSSEKVDVLRALGAEIVRTPTNARFDSPESHVGVA 207

CeCBS-2 472 LRLKHEIPGAVILDQYCNPGNPLAHYEETAEEILWDMGDRKIDLVVLGAGTGGTITGISRKIHERRPNAIVVGVDPNGSI 551

HsCBS 208 WRLKNEIPNSHILDQYRNASNPLAHYDTTADEILQQCDG-KLDMLVASVGTGGTITGIARKLKEKCPGCRIIGVDPEGSI 286

CeCBS-2 552 LTGPTT--GPAPDFYEVEGIGYDFIPGTLDEKSVDSWLKSDDKESFLMAREIIRTEGILCGGSSGCAVHYALEQCRKLDL 629

HsCBS 287 LAEPEELNQTEQTTYEVEGIGYDFIPTVLDRTVVDKWFKSNDEEAFTFARMLIAQEGLLCGGSAGSTVAVAVKAAQELQ- 365

CeCBS-2 630 PEDANVVVLLPDGIRNYLTKFLDDDWMKARGFL----------------------------------------------- 662

HsCBS 366 -EGQRCVVILPDSVRNYMTKFLSDRWMLQKGFLKEEDLTEKKPWWWHLRVQELGLSAPLTVLPTITCGHTIEILREKGFD 444

CeCBS-2 --------------------------------------------------------------------------------

HsCBS 445 QAPVVDEAGVILGMVTLGNMLSSLLAGKVQPSDQVGKVIYKQFKQIRLTDTLGRLSHILEMDHFALVVHEQIQYHSTGKS 524

CeCBS-2 ---------------------------

HsCBS 525 SQRQMVFGVVTAIDLLNFVAAQERDQK 551

**Fig. S1**

Protein alignment of *C. elegans* CBS-2 (CeCBS-2) and human CBS (HsCBS). Alignment was performed using Constraint-based Multiple Alignment Tool of NCBI. Conserved sequences are highlighted in red

**CeCBS-2** Met C L P T I K A F K T I L D A G S H R A T Q L V P L K K L K A E F Q L K S D I Y V K L E Y L N I S G S L E D R A A H R A F E Met S N G I Q R G D E V Y V T A G G S S A V S Y A T V A A V K G V R L T A H A P P G A F D Q V R T I L S T L G T K I V E L P V S N F R E A R L Q A D L L A K R R G V Y N L D G I C E K A A F L A N L E G T A P E I E K A G K T S A V V V P L D A G G A S A A A G I A A Y F L K D Q K T T V V G V R S S A S A S A S A P A P E T E V L L Q K Y K V E I R D A P E S Y T F T R H L I E T E G I Met A G P S S G A A V L E A I K L A K D L P A G S V V V V V L Met D G I R D Y L D A D W Met K V N G K K P I K P R E I F D P K V L D Y D P T K Met V G E W K S S S K F R P E R P L V L D S V L D A I G K T P L V K L Q H V P K A H G V R C N V Y V K C E F L N A G G S T K D R I A K K Met V E I A E K T G K P G A L T P G A T T L I E P T S G N T G I G L S L V A A V R G Y K C L I T Met P E K Met S K E K S T T L S V L G S T I V R T P N E A A F N S P S S H I G V A L R L K H E I P G A V I L D Q Y C N P G N P L A H Y E E T A E E I L W D Met G D R K I D L V V L G A G T G G T I T G I S R K I H E R R P N A I V V G V D P N G S I L T G P T T G P A P D F Y E V E G I G Y D F I P G T L D E K S V D S W L K S D D K E S F L Met A R E I I R T E G I L C G G S S G C A V H Y A L E Q C R K L D L P E D A N V V V L L P D G I R N Y L T K F L D D D W Met K A R G F L Stop

**ΔCeCBS-2**Met C L P T I K A F K T I L D A G S H R A T Q L V P L K K L K A E F Q L K S D I Y V K L E Y L N I S G S L E D R A A H R A F E Met S N G I Q R G D D S Stop D L R P E G A G L R S D E D G R G V E E F Q Q V Q A G K T T G S R L S P R C D R Q D A T G E A S A R S E G S W S Q V Q C L C Q Met R V P Q R G R L H Q R S N R Stop E N G R N C Stop E N R Q T R S T H P R S H H P H R A H L W Q H R D R P I P G R G G P W L Q Met P D H D A Stop E D V Q G E V D D A V G A R I H D C P D S Q R G G L Q Q P E L S H R S G S S L E A Stop D S G C G D S R S V L Stop S G E S T G T L Stop G D C G G D F V G Y G G S E D Stop S S G P W R R N R W Y H H R N L S E D P R A P S Q R D R R R R Stop P E W I N P D R P D D W T R S R F L R G Stop R D R L R L H P G D P G R K I S R F L A K I Stop Stop Stop G V I P D G S R D H Q N R G D L V W R I V G Met R G A L R V G A V P E A G S A G G C E C R C A A S R R D S E L S H Q I P R R R L D E S P R L L V

**Fig. S2**

Protein sequence prediction obtained using ExPASy online tool. CeCBS-2 is the translation of wild-type *cbs-2* nucleotide sequence. ΔCeCBS-2 is the translation of the nucleotide sequence of *cbs-2* lacking the 692 nucleotides corresponding to *ok666* deletion. Stop codon are highlighted in red. In ΔCeCBS-2 an early Stop codon is caused by *ok666* deletion.

**
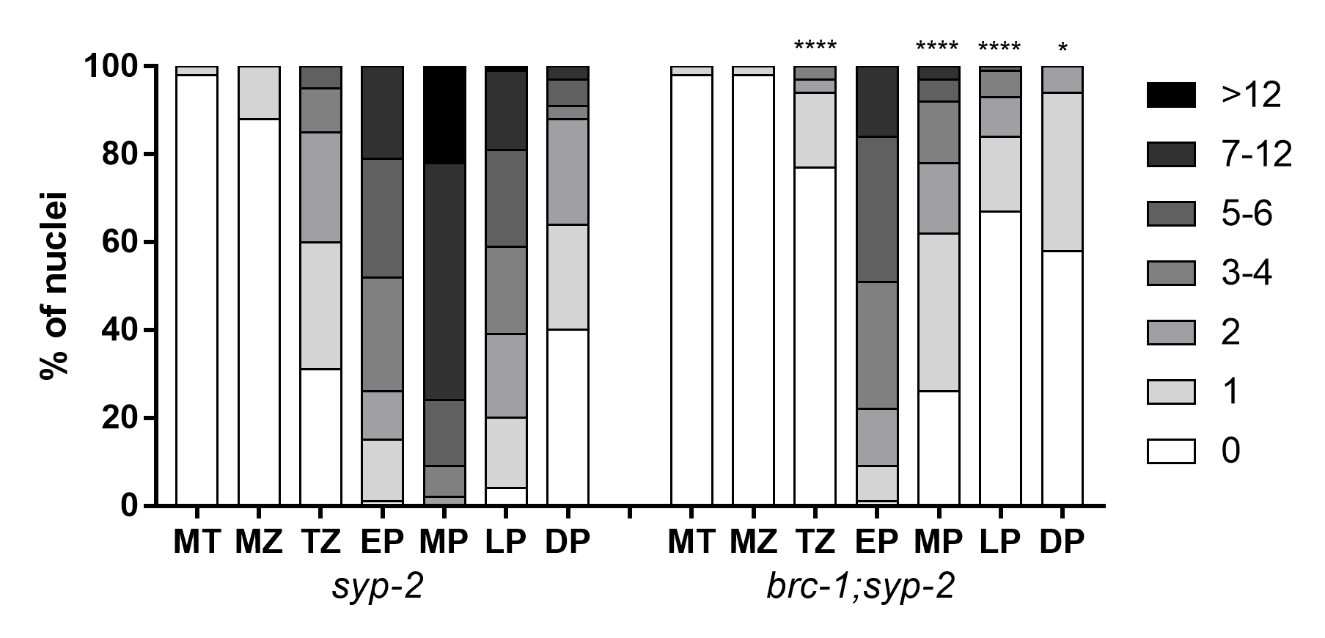
**

**Fig. S3**

Histograms represent quantification of RAD-51 foci in germline of the indicated genotypes. The y-axis represents the percentage of nuclei with the indicated number of foci. The x-axis represents the position (zone) along the germline. For each genotype an average of 100 nuclei from at least 4 different animals had been scored for mitotic tip, mitotic zone, transition zone, early, middle and late pachytene, while an average of 30 nuclei from at least 4 different animals had been scored for diplotene. Mann-Whitney *t*-test: * P=0.02; **** P< 0.0001. Statistical analysis indicated in Table S2

|  | **No** | **Hatched** | **No.** | **muv** | **vul** | **dpy** | **unc** | **L2/3**  **arrest** | **body cons.** | **tail- round.** | **head swol.** | **gon** | **sma** |
| --- | --- | --- | --- | --- | --- | --- | --- | --- | --- | --- | --- | --- | --- |
|  | **P0** | **progeby** | **Dev. Def.** |  |  |  |  |  |  |  |  |  |  |
| ***wt*** | 15 | 3986 | 1 |  |  |  |  | 1 |  |  |  |  |  |
| ***cbs-2*** | 20 | 3560 | 65 | 1 | 8 | 3 | 7 | 27 | 3 | 1 | 1 | 2 | 12 |
| ***wt* CDDP** | 12 | 659 | 34 | 1 | 4 |  | 6 | 17 |  | 1 | 1 |  | 4 |
| ***cbs-2* CDDP** | 11 | 241 | 28 |  | 5 | 1 | 2 | 16 | 2 | 1 |  | 1 |  |

**Table S1**

Post-embryonic developmental defects in untreated and CDDP (24-48 hrs) treated populations. Phenotype Abbreviations: muv (multi-vulva), vul (vulva-less), dpy (dumpy), unc (uncoordinated), L2/3 arrest (larval stage 2/3), body cons. (constriction at middle body), tail-round. (tail morphology variant), head swol. (the heat appears bloated), gon. (gonad development abnormal), sma (body size reduced)

| ***t*-test P - value** | Mitotic  tip | Mitotic zone | Transition zone | Early pachytene | Middle pachytene | Late pachytene | Diplotene |
| --- | --- | --- | --- | --- | --- | --- | --- |
| *wt vs cbs-2* | 0.34 | 0.18 | **< 0.0001** | 0.1 | **< 0.0001** | **< 0.0001** | **0.002** |
| *syp-2 vs cbs-2;syp-2* | 0.16 | 0.33 | 0.12 | 0.13 | 0.05 | 0.08 | **< 0.0001** |
| *syp-2 vs brc-1;syp-2* | 0.99 | 0.06 | **< 0.0001** | 0.86 | **< 0.0001** | **< 0.0001** | **0.02** |
| *cbs-2 vs cbs-2;syp-2* | 0.29 | 0.68 | **< 0.0001** | **< 0.0001** | **< 0.0001** | **< 0.0001** | 0.64 |
| *cbs-2:syp-2 vs brc-1;syp-2* | 0.25 | 0.25 | **< 0.0001** | 0.12 | **< 0.0001** | **< 0.0001** | **< 0.0001** |

**Table S2**

Statistical Analysis of RAD-51 foci in meiosis for the indicated genotypes

| **PRIMER** | **GENE** | **SEQUENCE (5’- 3’)** |
| --- | --- | --- |
| upper *cbs-2* ok666 | cbs-2 (F) | GAACTTGCTGGAACTCTTCC |
| lower *cbs-2* ok666 | cbs-2 (R) | TTGAAATTTCTCGGGGTACG |
| upper *fcd-2* (tm1298) | fcd-2 (F) | TCGCTCCGCCCTCTTTTCTA |
| lowe*r fcd-2* (tm1298) | fcd-2 (R) | CGACGAGCAGCTAACAACATTGG |
| upper *brc-1* (tm1145) | brc-1 (F) | TGTCGCATCGTCGGCATTAA |
| lower *brc-1* (tm1145) | brc-1 (R) | AATATAGGCACCGGCGGGGA |
| upper GFP | gfp [XR6] (F) | GCCCGAAGGTTATGTACAGG |
| lower GFP | gfp [XR6] (R) | CCCGGGCATTTGTATAGTTC |
| RT-upper*-cbs-2* | cbs-2 (F) | TGGTGCCGTTGAAGAAGC |
| RT-lower-*cbs-2* | cbs-2 (R) | CTCGTTGGATTCCGTTGG |
| RT-upper*-egl-1* | egl-1 (F) | TACTCCTCGTCTCAGGAC |
| RT-lower*-egl-1* | egl-1 (R) | CGAAGTCATCGCACATGC |
| RT-upper-*pmp-3* | pmp-3 (F) | GTTCCCGTGTTCATCACTCAT |
| RT-lower*-pmp-3* | pmp-3 (R) | ACACCGTCGAGAAGCTGTAGA |

# Table S3

List of all primers used in this study
